# Association of Chronic pain with Alzheimer’s‐related brain structure and plasma biomarkers in older men

**DOI:** 10.1002/alz.093994

**Published:** 2025-01-09

**Authors:** Tyler R. Bell, Carol E. Franz, Christine Fennema‐Notestine, Matthew S. Panizzon, Michael J. Lyons, Jeremy A. Elman, William S. Kremen

**Affiliations:** ^1^ University of California, San Diego, La Jolla, CA USA; ^2^ Boston University, Boston, MA USA

## Abstract

**Background:**

Chronic pain leads to tau accumulation and hippocampal atrophy in mice. Tau accumulation in the locus coeruleus (LC) precedes medial temporal accumulation in humans. Here we provide one of the first human studies examining the association of chronic pain with hippocampal volume, LC integrity, and Alzheimer’s Disease (AD)‐related plasma biomarkers. We predicted associations with rostral‐middle, but not caudal, LC because the former is more aging‐ and Alzheimer’s‐related.

**Method:**

Data were from 3 waves of the Vietnam Era Twin Study of Aging (VETSA) at average ages 56, 62, and 68. Chronic pain was defined as moderate‐to‐severe pain occurring >2 waves. At wave 3, 424 participants (mean age = 67.06) underwent LC‐sensitive MRI scans from which we calculated an LC contrast‐to‐noise ratio (LCCNR), an index of LC integrity. We measured hippocampal volume with traditional structural MRI. At wave 3, biomarkers were obtained from fasting blood draws taken the morning of the medical interview and cognitive testing, typically a day prior to the MRI exam. Immunoassays calculated levels of plasma total tau, beta‐amyloid (Aß) 40, Aß42, Aß42/Aß40 ratio, and neurofilament light (NfL). All biomarkers were log‐transformed and adjusted for site and storage time. Analyses accounted age, major health conditions, depressive symptoms, and opioid use.

**Result:**

Men with chronic pain had smaller hippocampal volume (ß = ‐0.24, p = .039) and lower rostral‐middle LCCNR (ß = ‐0.38, p = .004) but not caudal LCCNR (p = .750). Men with chronic pain also had higher levels of plasma total tau (ß = ‐0.32, p = .047), Aß42 (ß = .42, p = .033), and Aß40 (ß = .31, p = .032) compared to men without chronic pain. However, men with chronic pain did not differ from men without chronic pain on Aß42/Aß40 ratio (p = .979) or NfL (p = .313).

**Conclusion:**

Extending the animal model, these findings suggest that chronic pain is associated with tau accumulation and reduced structural brain integrity in regions affected very early in the development of AD. Increases in individual Aß measures associated among those with chronic pain but lack of association with the Aß42/Aß40 ratio may reflect non‐AD‐specific health‐related factors rather than brain amyloid, or alternatively, might be due to the relatively young age of the sample or the sensitivity of the assay.

